# The complexities of malaria disease manifestations with a focus on asymptomatic malaria

**DOI:** 10.1186/1475-2875-11-29

**Published:** 2012-01-31

**Authors:** Dolie D Laishram, Patrick L Sutton, Nutan Nanda, Vijay L Sharma, Ranbir C Sobti, Jane M Carlton, Hema Joshi

**Affiliations:** 1 National Institute of Malaria Research, Indian Council of Medical Research, Sector 8, Dwarka, New Delhi 110 077, India; 2 Center for Genomics and Systems Biology, Department of Biology, New York University, 12 Waverly Place, New York, NY 10003, USA; 3 Department of Zoology, Panjab University, Chandigarh 160014, India; 4 Department of Biotechnology, Panjab University, Chandigarh 160014, India

**Keywords:** Asymptomatic malaria, Host factors, Parasite factors, Transmission dynamics

## Abstract

Malaria is a serious parasitic disease in the developing world, causing high morbidity and mortality. The pathogenesis of malaria is complex, and the clinical presentation of disease ranges from severe and complicated, to mild and uncomplicated, to asymptomatic malaria. Despite a wealth of studies on the clinical severity of disease, asymptomatic malaria infections are still poorly understood. Asymptomatic malaria remains a challenge for malaria control programs as it significantly influences transmission dynamics. A thorough understanding of the interaction between hosts and parasites in the development of different clinical outcomes is required. In this review, the problems and obstacles to the study and control of asymptomatic malaria are discussed. The human and parasite factors associated with differential clinical outcomes are described and the management and treatment strategies for the control of the disease are outlined. Further, the crucial gaps in the knowledge of asymptomatic malaria that should be the focus of future research towards development of more effective malaria control strategies are highlighted.

## Background

Malaria remains a serious global health burden, with an annual incidence of 247 million cases and nearly one million deaths, most of which afflict children living in Africa [1]. Of the four human malaria parasite species, *Plasmodium falciparum *is reported to cause the highest morbidity and mortality. Young children with naïve immune systems [2] and pregnant women with potentially compromised immune systems are particularly vulnerable to this disease and so are considered to be the highest risk populations for malaria-related deaths. *P. falciparum *disease severity ranges from severe and complicated, to mild and uncomplicated, to asymptomatic [3,4]. Understanding the impact of *P. falciparum *on the human host across this range is critical for learning how to improve the management of the disease.

Generally, severe or complicated malaria has been at the core of epidemiological studies because it is the principal cause of malaria-related deaths. Researchers and clinicians have established diagnostic criteria based on the clinical manifestations upon disease onset, which has aided in forming an integrated approach to improving the management and treatment of severe malaria. Severe malaria is now defined by at least one of the following clinical manifestations: unrousable coma (caused by cerebral malaria), convulsions, malarial anaemia, haemoglobinuria, hypoglycaemia, metabolic acidosis (associated with respiratory distress), acute pulmonary oedema, acute renal failure, jaundice, circulatory collapse, hyperparasitaemia, high fever electrolyte disturbance, and/or spontaneous bleeding [4]. In areas of high transmission, this full spectrum of clinical severity is primarily observed in children; severe malaria is negatively correlated with age due to the development of exposure-related immunity in adults [5,6]. This is further supported by finding that frequent exposure to *P. falciparum *malaria in high transmission regions typically reduces the period of risk for severe malaria, while in lower transmission regions infrequent exposure extends this period of risk [5].

In contrast, individuals with mild or uncomplicated malaria typically present clinically with fever and perhaps one or more of the following symptoms: chills and sweats, headache, vomiting, watery diarrhea, anaemia, jaundice, and swelling of the spleen (splenomegaly), but do not generally have any of the features identified in severe or complicated malaria [4]. If properly diagnosed and treated, recovery success is high for patients with uncomplicated malaria (reviewed in ref [6]). Uncomplicated malaria also occurs in endemic areas and is likely associated with the development of some exposure-related immunity. For example, Gupta *et al. *[7] reported the development of clinical immunity to uncomplicated malaria after only one or two infective bites, highlighting the potential importance of strain-specific immunity.

Diagnosing asymptomatic malaria is not as straightforward due to the obvious lack of clinical manifestations and often subpatent (undetectable by microscopy) level of parasites [8]. Asymptomatic malaria is prevalent in malaria endemic regions and has become a serious cause for concern as efforts are increasing towards eliminating the parasite [9]. Particularly, subpatent malaria is still transmissible and will complicate elimination of malaria in high transmission regions. For example, a study in Senegal suggested that more than 90% of exposed individuals are likely infected with chronic asymptomatic malaria [8], a situation in which the majority of this population can then inadvertently act as a reservoir for malaria transmission.

For more than two decades, researchers have investigated the development of two types of immunity which may result in asymptomatic malaria: 1) an anti-disease immunity that allows one to carry parasite loads without symptoms; and 2) an anti-parasite immunity that may be responsible for the suppression of parasite loads after a certain age, which is likely a factor of exposure-related clinical immunity [10-12]. Interestingly, asymptomatic malaria is not only limited to regions of high transmission where exposure-related immunity is expected to develop; it has also been reported in the low transmission Amazonian regions of Peru, Brazil, and Columbia and also the Solomon Islands [13-21]. Exposure-related immunity may be achieved much earlier in life for individuals who live in low transmission regions due to predictably low parasite genetic diversity and few overlapping infections.

Few reports are available on the study of asymptomatic malaria caused by species other than *P. falciparum*. However, like asymptomatic *P. falciparum*, asymptomatic *Plasmodium vivax *malaria has been reported in a range of endemic settings. For example, the low transmission setting of Temotu Province, Solomon Islands [20] and the highly endemic malaria area of Rio Negro in the Amazon State, Brazil [22] both report significant presence of asymptomatic *P. vivax*. Another Amazonia study reports that the prevalence of "symptomless" falciparum and vivax malaria infections are 4-5 times higher than the symptomatic ones, with a significant correlation of "symptomless" malaria with older age groups [21]. Unfortunately, the reports above were limited to general prevalence surveys, without additional molecular analyses.

Though rare, cases of *Plasmodium malariae *and *Plasmodium ovale *asymptomatic infection have also reported. A case report of transfusion-transmitted *P. malariae infection *from an asymptomatic donor [23] suggests that *P. malariae *can be harbored asymptomatically. Supporting this, another study also reported a case of malaria in a Grecian woman due to *P. malariae *whose illness was reactivated after decades of latency [24]. Infection with *P. ovale *can also be asymptomatic [25]. However, these studies are mostly case reports and extensive longitudinal studies are lacking. Therefore, this review is focused on asymptomatic malaria due to *P. falciparum*.

The purpose of this review is to: (1) critically examine how asymptomatic falciparum malaria is defined; (2) describe potential relationships to transmission dynamics; (3) speculate on the potential roles of putatively important parasite genes; (4) review human genes that may contribute to susceptibility or resistance to infection; (5) discuss the management and treatment of asymptomatic malaria; and (6) discuss the gaps in knowledge and highlight areas of future research.

## The problem of defining asymptomatic malaria

A major obstacle in the study of asymptomatic malaria is the lack of standard diagnostic criteria. For example, infected individuals may be in a pre-symptomatic period with parasitaemia, and present with clinical manifestations at a subsequent date [26]. Alternately, studies that do not incorporate thorough clinical history surveys may not capture individuals that may have experienced symptoms for a brief period and then taken medication that suppressed parasitaemia and symptoms. The most widely-used criteria for diagnosis of asymptomatic malaria are presence of parasites in peripheral thick blood smears, an axillary temperature *<*37.5°C, and an absence of malaria-related symptoms [27-30]. Some studies do include other criteria, such as longitudinal follow-up and parasite quantification. Longitudinal follow-up is particularly important for differentiating between infections that appear asymptomatic at time of detection, but may become symptomatic after the initial detection [19-33]. Quantifying parasitaemia, rather than noting presence or absence of parasites, may also be an important consideration when diagnosing asymptomatic malaria. However, a universally standard parasite threshold for classifying an infection as asymptomatic has yet to be defined, as different studies use variable cut-off levels for parasite density [31-33]. Though the use of species-specific PCR possible is not always available in the field, or even perhaps practical for testing infections that are negative by microscopy, it is a powerful tool for finding asymptomatic malaria within a population. For example, Bottius *et al. *[8,34-36] found that as many as two-thirds of the microscopy-negative patients had subpatent levels of parasites determined by diagnostic PCR, indicating that almost the entire population was chronically infected with asymptomatic malaria. Examples of the varied diagnostic criteria used to classify malaria as asymptomatic by several different studies is presented in Table 1. For a more comprehensive list, see Additional file 1.

**Table 1:** A wide range of diagnostic criteria is used for defining malaria patients as asymptomatic

Country and Year	Criteria used for identifying asymptomatic malaria cases	Study subjects, sample size	Follow-up protocol and duration	**Ref**.
**Africa**				

Gabon, 2003	No clinical symptoms of malaria with a *P. falciparum *positive blood smear, asymptomatic for at least 5 days during follow-up.	Children 6 months to 10 years, N = 60	Examined once daily for 7 days thereafter, once every 2 days	[37]

Tanzania, 2006	Presence of *P. falciparum *on blood smear, axillary temperature of < 37.5°C, and no other symptoms or signs of malaria	Children 4-59 months, N = 127	No follow-up.	[33]

**South America**				

Brazilian Amazon, 2002	Individuals positive by microscopy, and/or positive by PCR; and individuals negative by microscopy that subsequently became positive by PCR.	All age groups, N = 172	Follow-up to day 10 and 60.	[21]

Colombia, 2008	Presence of microscopic asexual parasite stages of *P. falciparum*, *P. vivax *or *P. malariae *or of mixed infections in blood, which persisted for at least two weeks without causing any symptoms, or as the detection of parasite DNA by PCR on day 0 in people who remained asymptomatic during the follow-up period.	Individuals 2-78 years, N = 21	Follow-up on days 14 and 28.	[19]

**Asia**				

Papua, 2003	No fever history or treatment for malaria within the past week, no clinical evidence of malaria or other infection, no diarrhea, and no current pregnancy but both *P. falciparum *and *P. vivax *positive individuals	Adults > 16 years, N = 105	Supervised overnight at local health center. A third axillary temperature was recorded the following morning.	[38]

Indonesia, 2010	Presence of asexual *P. falciparum *or *P. vivax *parasitemia in the absence of fever (temperature ≤ 37.9°C) and of clinical signs or symptoms suggestive for malaria or another infectious disease.	Children 5-15 years, N = 381	No follow-up.	[27]

A more comprehensive list of studies is given in Additional file 1

## Transmission dynamics and asymptomatic malaria

### Infection prevalence

The transmission dynamics of malaria are complex. Even in endemic areas, transmission is not consistently stable; rather, it can be patchy and dependent on factors such as climate, the location of mosquito breeding sites, and areas of clustered human habitations, which serve as reservoirs of parasites for mosquito infection [39,40]. Asymptomatic infections often go undetected and untreated, resulting in a major source of gametocytes for local mosquito vectors [14]. Even in conditions where the possibility of re-infection is excluded, *P. falciparum *infection has been shown to persist asymptomatically in semi-immune individuals for more than 18 months [41]. Asymptomatic *P. falciparum *infections can also persist inter-seasonally in regions with seasonal transmission [42]. For example, even though the incidence of clinical malaria in Senegal is significantly lower during the dry season, a considerable proportion of the population remain parasitaemic throughout the year [29,43,44]. This reaffirms that malaria transmission can persist in areas where symptomatic cases have been monitored and treated. This model has driven presumptive intermittent anti-malarial treatment strategies for the treatment of asymptomatic individuals, regardless of their infection status, to reduce the disease burden.

In areas with annual malaria transmission, asymptomatic *P. falciparum *parasitaemia is common among immune inhabitants [45,46] and a large proportion of individuals always harbor malaria parasites without any associated clinical symptoms [47]. A positive correlation between high transmission and high asymptomatic prevalence has been reported in Nigeria, Senegal, Gabon and Amazonian regions of Brazil [8,34-36]. Similarly, an increase in the prevalence of asymptomatic vivax malaria has been noted in high transmission regions, including Thailand, Sri Lanka, and Brazil [48,49]. High prevalence rates of asymptomatic malaria within areas of high transmission might be due to exposure-related immunity. Exposure-related immunity occurs when individuals who are frequently exposed to parasites for long periods of time eventually develop immunological memory that suppresses infection. Paradoxically, asymptomatic malaria has also been reported in low transmission areas [15-21]. Two studies in the low transmission Peruvian Amazon demonstrated that high parasite exposure is not necessary for the development of B-cell memory or long-lasting antibody titres [50,51]. Though exposure is thought to help maintain immunological memory, other factors besides the transmission intensity may be associated with the prevalence of asymptomatic malaria within different regions.

### Mosquito infectivity

The production of gametocytes within an infected human host is essential for transmitting the parasite to the mosquito. Understanding the variables that are involved in gametocyte production within the human host, such as the host immune response to parasites, anti-malarial drug treatment, and parasite genetic diversity, and correlating these with the presence or absence of symptoms, is layered with complexity. For example, some studies have indicated that the quantity of gametocytes may impact the level of mosquito infectivity [52]; however, asymptomatic carriers of sub-microscopic gametocyte densities are also infectious to the mosquito host, as found in some high transmission regions [52,53]. It seems that the quality, not the quantity, of gametocytes may be more important for infectivity [53-59]. Anti-malarial drug treatment has been shown to negatively impact gametocyte quality, by reducing gametocyte infectivity upon a blood meal [60]. In turn, this complicates controlled investigations of disease severity and gametocyte infectivity of the mosquito host, since more often than not, epidemiologists rely on association studies between gametocyte density or gametocyte ratios and disease severity to extrapolate a possible relationship.

There is evidence that patients with severe anaemia have higher levels of gametocytes [61], which may be the result of environmental cues released in the blood upon parasite-induced haemolysis, or alternatively may represent an artificial inflation due to the decreased number of circulating red blood cells. An earlier study also provided the evidence of this important relationship between the development of patent *P. falciparum *gametocytaemia and transmission potential [62]. The study suggested that the sexual stages are not pyrogenic and are commonly seen in subjects without fever, in whom the asexual parasitaemia has fallen below the pyrogenic threshold. This is supported by finding in their study that patients who were afebrile and presented with a low asexual parasitaemia were 2.7 times more likely to present with a patent gametocytaemia compared to those without both of these factors. Once these sexual stages have been formed, they are harbored and may persist for up to three weeks, imparting a persistent ability to infect mosquitoes [63,64].

A study in Thailand investigating mosquito infectivity differences between febrile and asymptomatic patients reported that febrile patients were more infective than asymptomatic individuals to mosquito hosts [65]. This was recapitulated in a study in Amazonian natives, which reported that highly parasitaemic, symptomatic patients were more infective to mosquitoes than asymptomatic patients with submicroscopic parasitaemia [14]. However, the authors reported that asymptomatic infections were 4-5 times more prevalent and infective for longer periods of time post-treatment. In contrast, Gouagna *et al. *reported that parasites from asymptomatic individuals in an endemic region of western Kenya were more infective to mosquitoes than parasites from symptomatic individuals. The authors concluded that this was a function of both the greater gametocyte abundance in asymptomatic individuals and a higher quality of infectious gametocytes [66,67].

These observations of asymptomatic malaria in low and high endemic areas highlight the importance of carrying out active surveys for the identification of asymptomatic carriers. The treatment of the infective parasite reservoir of asymptomatic individuals may be an important intervention strategy, and if this reservoir is greatly reduced it will provide a positive impact on the intervention of disease transmission.

## Role of the malaria parasite in asymptomatic malaria

It is not clear why some *P. falciparum *infections are symptomatic while others are asymptomatic, but parasite factors are likely to be involved [68]. Malaria parasites influence disease outcome through factors that include parasite density, rosetting and sequestration, toxin production, and genetic diversity including expression of virulence and immune evasion genes such as the *var *(variant antigen receptor) gene family.

### Parasite density

The precise relationship between parasite density and disease severity remains unclear, as the density of sequestered parasites and circulating parasites varies greatly depending upon the stage and synchronicity of the infection [69]. In general, high parasitaemia has been associated with increased disease severity; however, this is not always the case as peripheral parasitaemia does not always accurately reflect the number of parasites due to sequestration. For example, Silamut *et al. *reported cerebral malaria as the cause of death in aparasitaemic individuals in autopsies following effective antimalarial treatment [70]. A study in the Solomon Islands showed that both *P. vivax- *and *P. falciparum-*infected asymptomatic individuals tend to have low and submicroscopic parasite densities [20]. Another study suggested the association of asymptomatic parasitaemia of higher parasite density with a higher risk of symptomatic malaria [71].

A study investigating the relationship that parasite density has on platelet count showed that malaria-infected children with thrombocytopenia (decreased platelet count) were younger, had higher parasitaemia, lower hemoglobin levels, an increased mean platelet volume, and exhibited platelet aggregation [72]. Similarly, a cross-sectional study on Nigerian children with asymptomatic malaria showed that malaria parasites cause a significant reduction in platelet counts with more pronounced reduction in children under 5 years of age [73]. Therefore, thrombocytopenia is not only a feature exhibited by acute malaria, but also a potentially useful indicator for monitoring asymptomatic children in high transmission areas.

### Strain diversity

Various studies have investigated the genetic diversity of *P. falciparum *and its association with the development of clinical symptoms. There is strong evidence that immunity to malaria is specific to the particular strain eliciting the host response, enabling an individual to resist infection by that particular strain, but not by heterologous ones; this has been termed 'strain-specific immunity' [74-76]. The development of strain-specific immunity might then be somewhat responsible for decreased disease severity, including asymptomatic malaria, in populations where exposure is moderate to high.

Other studies have sparked an investigation of some symptom-specific molecular characteristics based on polymorphisms of surface antigens that have been identified in isolates collected from asymptomatic, uncomplicated, and severe malaria cases across various geographic regions [77-79] (see Additional file 2). Such polymorphic surface antigens include the merozoite surface protein (MSP) family, apical membrane antigen 1 (AMA1), erythrocyte binding antigen-175 (EBA-175), and knob-associated histidine rich protein (KAHRP). Specific antigenic polymorphisms may allow the parasite to evade the immune response in patients with chronic infection and thus it may lead to their survival in the human host until transmission becomes possible.

### Merozoite surface proteins

Several studies have investigated the association of *msp-1 *and *msp-2 *with clinical severity of disease. For example, a study conducted to examine the relationship between the genetic diversity of *msp-1 *block 2 of *P. falciparum *and clinical severity of malaria in Nigerian children showed that the presence of K1 and MAD20 alleles were significantly associated with asymptomatic malaria and consequently reduced the risk of developing symptomatic disease [80]. Yet, it is possible that frequency of exposure to these allelic families may also be a factor. Ariey *et al. *[81] found that the association of a specific *msp-1 *allele (K1) with a specific *var *gene (*var-D*) was overrepresented among patients with severe compared to mild disease, and this genotype combination was consistently observed in the most severe clinical cases. However, investigations of genotype associations with disease severity need to be expanded to include all categories of disease, including asymptomatic malaria. Further, genetic diversity should be longitudinally monitored to ensure that such clinical associations are maintained and not biased by cross-sectional selection of isolates.

There are conflicting reports regarding the differential distribution of alleles within particular genes (such as *msp-2*) according to clinical status [82,83]. The FC27-like genotype of *msp-2 *was shown to be twice as likely to be found in symptomatic cases than in asymptomatic cases [83], providing evidence that specific variants of *msp-2 *may be associated with the morbidity of malaria. A similar association was reported in Papua New Guinea, where the FC27 allele was linked to increased disease severity, however, this study did not consider asymptomatic cases [84]. Conversely, another study reported that there was no association between FC27 or 3D7 alleles of *msp-2 *and malaria symptoms [85]. However, further longitudinal studies in different geographical settings with standardized collection and genotyping methods will be required to clarify these findings.

### Apical membrane antigen 1

Multiple lines of evidence indicate that polymorphisms in the *P. falciparum *AMA1 domain I result from selective pressures exerted by protective host immune responses [86]. In a study in Papua New Guinea, a pattern of geographical diversity and the particular substitutions found were suggestive of strong constraints acting on the evolution of AMA1 at the population level. In addition, differences between the sequences of AMA1 domain I from symptomatic and asymptomatic infections implicate AMA1 as a possible determinant of the morbidity associated with a particular *P. falciparum *strain [86].

### Erythrocyte binding antigen 175

Studies on the distribution of EBA-175 genotypes suggest that this gene plays a role in different clinical outcomes. Genomic studies of two *P. falciparum *strains, namely FCR-3 and CAMP, revealed two highly dimorphic segments in region III, which is located in the central part of the gene [87]. Dimorphism is referred to as the F-fragment in the FCR-3 strain and the C-fragment in the CAMP strain. In a study, the CAMP(C) and FCR-3(F-) genotypes of the EBA-175 were encountered in both symptomatic and asymptomatic patients, but the FCR-3 genotype predominated regardless of clinical status and the sampling period since the FCR-3(F-) genotypes were simply more prevalent in the region [22]. However, the prevalence of mixed C-/F- infection was far higher in symptomatic than in asymptomatic children. This study showed that mixed C-/F- infection is associated with clinical malaria and may have therapeutic implications [32].

### Virulence genes

An important aspect of *P. falciparum *virulence is the ability of infected erythrocytes to sequester and obstruct the microvasculature of different organs. Cytoadhesion to endothelial cells is mediated by electron-dense elevations of the parasite membrane referred to as knobs. Knobs consist predominantly of the knob-associated histidine-rich protein (KAHRP), which cluster on the cytoplasmic face of the knob membrane [88]. KAHRP is required for knob formation and has also been used as a marker because of its role in pathology [89]. In one study, three different alleles (340 bp, 370 bp and 400 bp) were found in mild cases, but only two forms (340 bp and 370 bp) were observed in severe cases [90]. Studies on the association of these KAHRP alleles with severity need to be extended to asymptomatic malaria.

Many gene expression studies in *P. falciparum *have focused on the diverse *var *gene family, encoding various forms of erythrocyte membrane protein 1 (PfEMP1), which is involved in cytoadherence of RBCs (red blood cells) to endothelial cells [91]. Switching of PfEMP1 isoforms through differential expression of members of the *var *multigene family is thought to facilitate evasion of the host immune response [91,92]. Most *var *genes fall into three major groups: *var *A, *var *B, and *var *C. Emerging evidence suggests that specific expression of these groups has clinical relevance and that major differences in the transcription profile of *var *gene expression may differentiate disease severity [33,93]. For example, *var *group C transcript levels were increased in asymptomatic cases, whereas transcripts of *var *group A and B were abundant in patients with severe malaria [33]. Another study reported that *P. falciparum *isolates from cerebral malaria patients are significantly more likely to transcribe *var *genes with DBLα1-like domains, characteristic of Group A and Group B/A (an intermediate group of *var *gene), than the isolates from patients with non-severe hyperparasitaemia (*i.e.*, high parasitaemia with no symptoms or signs of severe disease) [94]. Recent studies on *var *gene expression and malaria severity have yielded conflicting results [33,93,95,96]. These studies are complicated by extensive variation in the *var *gene transcription and the phenotypes displayed by circulating parasites, which may be different to those sequestered, as in severe malaria. Therefore, comparing the repertoire of genomic and expressed *var *genes with a particular malaria outcome will be helpful to understand the role of this gene family in asymptomatic malaria.

### Other factors that influence malaria outcomes

The formation of rosettes may influence the severity of malaria by causing distinct patterns of sequestration with different pathogenic consequences [68]. Parasite toxins such as glycosylphosphatidylinositol (GPI) anchors and haemozoin have been proposed to drive shock-like syndromes during infection [97,98]. However, the roles of these factors have not been reported in asymptomatic cases.

Intraleucocytic malaria pigment found in neutrophils is suggested to be a better indicator of disease severity than the peripheral parasite count [99,100]. Nguyen *et al. *[100] first reported an increase in the proportion of malaria-pigment containing neutrophils and monocytes in severe malaria patients. Later, Amodu *et al. *[99] confirmed that there was an unambiguous rise in the proportion of malaria pigment-containing neutrophils as severity increases across a range of disease outcomes, from uninfected to severe malaria cases. Amodu *et al. *[99] also reported that the proportion of pigment-containing monocytes did not differ significantly between mild malaria, asymptomatic malaria, and no malaria groups; however, the severe (cerebral) malaria group had a higher median value than the other three groups. Further, a case-control investigation [101] expanded and validated the finding that monocytes and neutrophils are markers of disease severity, but unfortunately asymptomatic malaria cases were not assayed in this study.

## Role of human host factors in clinical outcomes

Studies on the association of the roles of host factors such as genes of the immune system, RBC polymorphisms or disorders, circulating levels of immunoglobulins and cytokines, and pregnancy should not be limited only to symptomatic malaria, but also include asymptomatic malaria. To date this has usually not been the case; the role of host factors in asymptomatic malaria largely remains a field ripe for inquiry.

### Polymorphisms of immunologically relevant genes

Significant correlations between SNPs (single nucleotide polymorphisms) in regulatory or coding regions and severe malaria have been reported for several immunologically relevant genes, including cluster for differentiation-40 ligand (CD40L), Fc gamma receptor II (FcγRII), complement receptor 1 (CR1), tumor necrosis factor α (TNF-α), interleukins -4, -12, and -13 (IL-4, IL-12, IL-13), intracellular cell adhesion molecule-1 (ICAM-1), CD-36, platelet endothelial cell adhesion molecule-1 (PECAM-1), toll-like receptor (TLR), and mannose binding lectin 2 (MBL2)[102-115]. However, the association between a particular SNP and response to infection is highly dependent on ethnic background, *i.e*., an association demonstrated for one ethnic group may not be the same for a population that differs both in genetic background as well as frequency of exposure to infection [116]. Thus, disease correlation with certain SNPs inferred from studies of African or Caucasian populations may or may not yield the same results in ethnically divergent or admixed populations. For example, allelic and genotypic frequencies of interleukin-10 (IL-10-1087 A/G) and in IL-4-590 C/T polymorphisms have been reported to vary based on inter-ethnic differences [117]. A study conducted in Gabon reported no statistically significant association between MBL, TNFα-308, or NOS2 polymorphisms and asymptomatic malaria [118]. However, this may not be true for other geographical regions of the world. Studies on the genetic differences between populations may provide a better understanding of the role of these genes. A summary of the variants and polymorphisms of the erythrocytic and immunologically relevant genes of the human system involved in malaria susceptibility and resistance is given in Additional file 3.

### RBC polymorphisms or disorders

Several RBC polymorphisms, like G6PD deficiency [118,119], haemoglobin variants [120,121], ABO blood group antigen [122], ovalocytosis [123], and polymorphisms in complement receptor 1 [124], have been shown to provide at least some protection against severe malaria, suggesting the possibility of coevolution between parasite and host. Such protective roles are much less clear for mild malaria and for asymptomatic malaria [125].

However, research on G6PD polymorphisms has expanded in the last decade to include a range of disease outcomes. For example, G6PD A^- ^heterozygosity in females confers protection against all forms of malaria, including the asymptomatic form [118]. The mechanism of this protection may be because the parasite in G6PD A^- ^heterozygous female host must cycle between G6PD A^- ^and G6PD wild type erythrocytes and may fail to adapt to the G6PD A^- ^environment [126].

The global distribution of ABO blood group antigens reflects natural selection by various pathogens. There is strong evidence that O blood group provides protection against malaria by a mechanism of reduced rosetting and sequestration [127], while other reports have found an association between presence of blood group A with higher incidence of severe malaria [128,129]. A study that associated the blood groups of children with malaria disease outcome, found a high prevalence of asymptomatic malaria in children with the blood group O antigen, when compared with those without this antigen [121]. This study suggested that the blood group O provides protection against clinical forms of malaria. However, the association in this study was weak (*p *= 0.05). Further evidence for such protective effects must not only consider the distribution of blood groups, but also other explanations, such as the anti-rosette formation effect associated with blood group antigens [130].

### Immunity due to circulating levels of immunoglobulins and cytokines

Different clinical outcomes during malaria infection may be due to differences in the host immunity level. Acquisition of natural immunity to malaria has been observed in high and stable (*i.e.*, intense and constant over months and years) malaria transmission areas. This immunity acquired due to repeated exposure reduces the risk of both severe and mild malaria. Therefore, asymptomatic malaria could be a consequence of natural immunity. However, in low transmission areas in South America clinical immunity is found to develop in underexposed individuals, resulting in asymptomatic malaria [50]. Hence, it could be possible that a person in a low transmission area develops immunity faster because: (1) there is less antigenic diversity circulating in the regions and/or (2) there are less infections to overwhelm the immune response [131,132].

Malaria infection induces polyclonal immunoglobulin production the proportion of which determines protection against the blood stages of *P. falciparum*. Evidence suggests that antibody-dependent mechanisms play an important role in the reduction of parasitaemia and this alleviates clinical symptoms, as demonstrated by the passive transfer of hyper-immune immunoglobulin G (IgG) [133,134]. Among the various IgG isotypes, cytophilic antibodies IgG1 and IgG3 have been consistently correlated with uncomplicated malaria and even offer protection, while IgG4 does not protect against malaria [135-139]. Infections have also been associated with elevations in total IgE, with higher levels detected in cerebral *P. falciparum *malaria than in uncomplicated mild malaria [140,141]. The role of anti-parasite specific IgE is, however, controversial. Some studies have observed higher antimalarial IgE levels and functional activity in asymptomatic and uncomplicated malaria groups than in severe or cerebral malaria groups [30]. Additionally, high anti-*P. falciparum *IgE levels have been associated with a reduced risk of developing clinical malaria [142]. This supports a theory of IgE antibodies having a role in protection against disease. Results from others studies suggest that high levels of parasite-specific IgE are observed in patients with severe malaria, which indicates a putative role in pathogenesis [143-146].

One of the most challenging issues with studying malaria immunology is that individuals at risk for malaria infections are typically at risk for other parasitic or non-parasitic diseases. Distilling the immunological response induced by malaria apart from other infectious diseases is not straightforward. For example, co-infection of *Plasmodium *parasites with soil transmitted helminthes or Hepatitis B Virus (HBV) has been reported in several studies. Helminth infections may alter susceptibility to clinical malaria by changing the T-helper1/T-helper2 (Th1/Th2) balance [147,148], thereby affecting immunoglobulin levels. These findings are based on the assumption that helminth infections induce a strong and highly polarized immune response [149], which has been suggested to help facilitate the acquisition of immunity to malaria. In contrast, HBV when co-infected with *Plasmodium *influences the malaria burden by stimulating an increased inflammatory response. *Plasmodium*-infected individuals with HBV infection were more likely to be asymptomatic with lower levels of parasitaemia and a decreased inflammatory cytokine profile [150]. Thus, the host's immune system is modified by other co-infecting organisms thereby complicating our understanding of malaria clinical outcome.

Age is considered one of the most important factors that correlate with protective immunity in malaria endemic areas. Infections among non-immune individuals invariably result in clinical symptoms, and often lead to death in young children if untreated [11]. Young children are most susceptible to malaria infections and disease onset as they have not yet acquired clinical immunity. In endemic areas, adults and older children have a lower prevalence of malaria infection and lower incidence of clinical malaria [151]. These individuals acquire immunity from severe malaria episodes during childhood [7] and therefore as an adolescent or adult they are more likely to develop uncomplicated or asymptomatic malaria than severe malaria [6]. Severe cases are typically found in adults if they are non-immune or have not encountered malaria before [152,153].

In adult individuals, the acquired immunity or protection which is thought to keep their malaria asymptomatic or uncomplicated could be due to increased frequency of both type 1 and type 2 cytokine-producing T cells [154]. Cytokines are involved in both protection from, as well as the pathogenesis of, malaria infections. Elevated levels of circulating cytokines such as interleukin-6 (IL-6), IL-12, IL-1, and IL-10, as well as high circulating levels of TNF-α, all appear to have some correlation with the severity of the disease [111]. In falciparum malaria, cytokines may stimulate beneficial immunological responses by inducing acute phase responses, inhibiting parasite growth, and clearing vascular parasites and debris. Inflammatory responses mediated by interferon gamma (IFN-γ) in the interleukin 12 (IL-12) and 18 (IL-18) dependent manner, seems to be crucial for the control of parasitaemia through the induction of tumor necrosis factor (TNF) and the enhanced release of anti-parasitic reactive nitrogen and oxygen radicals. TNF and INF-*γ *stimulate neutrophils in order to increase parasite destruction [155].

### Pregnancy

Pregnant women are more susceptible to malaria infection, a phenomenon especially apparent during the first pregnancy [156]. In successive pregnancies, a decrease in intensity of infection has been observed and attributed to the acquisition of antibodies against variant surface antigens (VSA) such as PfEMP-1 (*P. falciparum *Erythrocyte Membrane Protein-1), rifins, and stevors. These antigens are expressed on parasitized RBCs infecting the placenta and are most commonly referred to as Variant Surface Antigens of Pregnancy Associated Malaria (VSA-PAM) [157,158]. The immunological relevance of these biological factors associated with asymptomatic malaria needs to be established. Studies on the evaluation of cytokine and growth factor in asymptomatic pregnant women showed an association between increased plasma concentrations of IL-10 and G-CSF [159].

## Treatment and management of asymptomatic malaria

Drug therapy has often been linked to the development of gametocytaemia, as environmental pressure caused by the targeted destruction of the asexual stage parasites stimulates the production sexual stage parasites (reviewed in [160]).Though this may be true, there is also mounting evidence, found in studies like Bousema *et al. *[161], which suggests that the production of gametocytes may be unrelated to drug therapy. In this study, Bousema *et al. *[161] investigated gametocytaemia in a cohort of untreated, asymptomatic children (less than five years old). This study reported that despite a lack of treatment, a significant proportion of these children had quantifiable gametocytes. Therefore, asymptomatic individuals have the potential to develop gametocytes even in the absence of drug therapy and this pool may serve as reservoir of disease transmission.

Since the transmission of malaria parasites from humans to mosquitoes requires the presence of gametocytes, any strategy that interferes with the development or persistence of gametocytes should help to interrupt transmission. In most malaria endemic areas, the majority of parasite carriers are asymptomatic [162] and these carriers typically do not seek medical treatment. Therefore, asymptomatic individuals carrying gametocytes remain available as a reservoir for transmission by mosquitoes, contributing to the persistence of malaria transmission within local populations [14,161].

The identification and management of asymptomatic carriers has become a new and increasingly important challenge for malaria control programs. One challenge, in particular, is to reform the development of novel anti-malarial drugs to include those that explicitly target transmissible sexual stages. At the present, the majority of anti-malarial drug treatments on the market target the asexual blood stage of *P. falciparum*. Treatment regimens containing artemisinin and/or its derivatives are reported to lower gametocyte carriage, and reduced infectivity among treated individuals [163-165]. Artemisinin combination therapy (ACT), which is advocated as the first-line of anti-malarial treatment, and has been reported to be efficient in reducing even submicroscopic levels of gametocytes [55,164,166,167]. Intermittent preventive treatment (IPT), the administration of a full course of an anti-malarial treatment to a population at risk at specified time points regardless of the infection status of individuals, has been proposed as a method of treatment for asymptomatic individuals to reduce transmission of disease. Artemether-lumefantrine, an ACT drug currently available on the market, has been suggested as a candidate for IPT treatment of asymptomatic carriers [168]. The short half-life of lumefantrine makes it an ideal treatment strategy because of concerns for developing resistance to ACT. Pharmacokinetic determinants of resistance selection indicate that this drug has a considerably shorter "window of selection," when compared to other artemisinin companion drugs, such as mefloquine [169].

Apart from selection for drug resistance, there is an additional risk of the persistence of submicroscopic gametocytes even after treatment, allowing for post-treatment malaria transmission [166]. Primaquine, a widely used drug for the treatment of *P. vivax *malaria, actively clears submicroscopic *P. falciparum *gametocytes [170-172], but the possible haemolytic effects of primaquine in relation to G6PD deficiencies must be considered before marketing this drug for the treatment of gametocytes. Reports indicate that artesunate (a derivative of artemisinin) may predominantly inhibit gametocyte development, while primaquine may accelerate its clearance [172]. However primaquine, when given in combination with sulphadoxine-pyrimethamine and artesunate, was found to be both safe and highly efficient in clearing *P. falciparum *gametocytes and asexual parasites detected by microscopy [173] as well as submicroscopic gametocytes [174]. As part of surveillance and control strategies for malaria, the systematic diagnosis or identification and treatment of asymptomatic carriers could reduce the pool of parasites available for the infection of mosquitoes.

The transmissibility of submicroscopic parasites within asymptomatic malaria infections may help to drive the persistence of malaria within endemic regions. The use of antimalarial drug treatment (particularly ACTs), in combination with insecticide treated nets (ITNs), long-lasting insecticidal nets (LLINs), and indoor residual spraying (IRS) is perhaps the most aggressive method for reducing the malaria burden in endemic regions. A recent study by Aregawi *et al. *[175] using ACT in combination with ITNs/LLINs and IRS demonstrated a 76% reduction in slide positivity for all age groups over a 4 year intensive intervention effort. Singling out the mosquito vector by undertaking comprehensive entomological and ecological studies is necessary for fully understanding malaria transmission dynamics, *i.e*. the identification of vector breeding and resting sites, the impact of climate change and temperature variation on vector survival and capacity, and also insecticide resistance.

## Perspectives and future research

The history of research on asymptomatic malaria dates back to 1900 when Robert Koch first identified such cases in a study among patients in Papua New Guinea [176]. Yet, asymptomatic malaria has rarely been a major research focus. Since individuals with asymptomatic malaria present with no symptoms, there are inconsistencies in defining, difficulties in diagnosing, and a general lack of urgency to investigate this particular disease outcome. In some sense, asymptomatic malaria has become the "forgotten" malaria, although recently asymptomatic malaria has become accepted as a major hurdle for malaria elimination, as infected hosts serve as silent reservoirs. The treatment of asymptomatic carriers as part of routine surveillance strategies has the potential to make a significant contribution to the reduction of malaria in endemic regions.

Scaling up efforts to improve the characterization of asymptomatic malaria in endemic regions and establishing a standard case definition for this malaria disease outcome is a priority. To help facilitate this in field studies, longitudinal active case detection methods, improved epidemiology questionnaires, and advanced epidemiological modeling must become universal components in all epidemiological studies. Concerted efforts should also be directed towards the development of highly sensitive, cheap and easily obtained rapid diagnostic detection kits, which can be used to detect parasitaemia at submicroscopic densities [177]. Identifying the extent of the problem is a major challenge.

Secondarily, the underlying causes of asymptomatic malaria must be fully investigated. It is important to expand beyond the traditional association and correlation analyses to include more comprehensive molecular methods. If detection efforts are improved, then it will be possible to discriminate between the host and parasite roles in asymptomatic infection. Consequently, parasite population diversity studies will become more thorough, the association of parasite virulence genes will become more relevant, and the malaria field will have a better sense of how to interpret and manage different disease outcomes. Importantly, information on host genetics and immune responses in association with asymptomatic malaria are severely lacking, but could provide a wealth of knowledge on the management of disease and the roles that they play in mounting different responses to infection.

## Competing interests

The authors declare that they have no competing interests.

## Authors' contributions

DDL and PS mined the literature. DDL, PLS, NN, VLS, RCS and JMC wrote the manuscript. HJ contributed to initial discussions and planning. All authors with the exception of HJ (deceased) read and approved the final manuscript.

## Supplementary Material

Additional file 1 **Diagnostic criteria for defining malaria patients as asymptomatic**. The list represents a snap-shot of some of the studies of asymptomatic infections world-wide. Click here for file

Additional file 2 **List of *P. falciparum *genes reported to be associated with different clinical outcomes of malaria**. (DOC 78 kb). Click here for file

Additional file 3 **List of human genes reported to be associated with different clinical outcomes of malaria**. A. Human gene polymorphisms B. Human blood disorders. Click here for file
