# Supplementary material for: The complexities of malaria disease manifestations with a focus on asymptomatic malaria
Source: Malar J. 2012 Jan 31;11:29. doi: 10.1186/1475-2875-11-29 (PMC3342920; doi:10.1186/1475-2875-11-29)
Supplement: Additional file 1 — Diagnostic criteria for defining malaria patients as asymptomatic. The list represents a snap-shot of some of the studies of asymptomatic infections world-wide. [file 1475-2875-11-29-S1.DOC]

**Additional file 1. Diagnostic criteria for defining malaria patients as asymptomatic.** The list represents a snap-shot of some of the studies of asymptomatic infections world-wide.

| **Country and Year** | **Criteria used for identifying asymptomatic malaria** | **Study subjects, sample size** | **Follow-up protocol and duration** | **References** |
| --- | --- | --- | --- | --- |
| **Africa** | | | | |
| Nigeria, 1998 | Individuals with *P. falciparum* trophozoites in blood smear but without malaria symptoms, no signs of any other illness, and normal findings on clinical examination. | Children  4 to14 years,  N=146 | No follow-up. | [1] |
| Gabon, 2003 | No clinical symptoms of malaria with a *P. falciparum* positive blood smear, asymptomatic for at least 5 days during follow-up. | Children  6 months to 10 years, N=60 | Examined once daily for 7 days for the presence of parasites on thick blood smears; thereafter, once every 2 days until clinical symptoms become apparent. | [2] |
| Gabon, 2003 | Parasite density ≤800 parasites/µl of blood, and no symptoms in either the two weeks prior to or the days following blood collection. | Children  7 to 19 years,  N =158 | Specific duration not mentioned. | [3] |
| Gabon, 2006 | Axillary temperature <37.5 °C and the absence or the presence (under 5000 parasites/µl of blood) of *P. falciparum* asexual on thick blood smear. | Children  6 to 114 months,  N=180 | No follow-up. | [4] |
| Gabon, 2006 | Axillary temperature <37.5°C on day of recruitment, no history of fever in the 24 hours before or the week after recruitment, and free of sickle cell disease; <5000 asexual blood stage parasites per microliter of blood. | Children  6 to 15 years,  N=116 | One week. | [5] |
| Gabon, 2007 | No clinical manifestation of malaria but a *P. falciparum*-positive thin blood smear. | Children  0.1 to 6 years,  N=135 | No follow-up. | [6] |
| Kenya, 2005 | Afebrile and no symptoms of malaria regardless of microscopy results for *P. falciparum.* | Children  10 to 31 months,  N=259 | No follow-up. | [7] |
| Kenya, 2009 | No detected episode of febrile malaria but asymptomatic *P. falciparum* parasitemia at the second cross-sectional bleed after 3 months | Children  1 to 6 years,  N=381 | Every week for three months. | [8] |
| Kenya, 2009 | A positive *P. falciparum* blood smear with no signs of malaria and parasitemia <10,000 parasites/*μ*L. For a more strict classification, individuals with fever (with or without parasites) at the time of or within 1 week after the survey and afebrile children with >10,000 parasites/*μ*L were excluded. | Children  0 to10 years, N=1285 | One week. | [9] |
| Senegal, 2008 | Presence of *P. falciparum* parasitemia on blood smear, without any clinical signs of malaria such as fever, or history of fever | Individuals  2 to 18 years,  N=1356 | Specific duration not mentioned. | [10] |
| Tanzania, 2004 | No record of a clinical malaria episode 4 weeks before and 1 week after the survey but a *P. falciparum*-positive thick blood smear. | Individuals between  1–84 years,  N=700 | One week. | [11] |
| Tanzania, 2006 | Presence of *P. falciparum* on blood smear, axillary temperature of <37.5°C, and no other symptoms or signs of malaria | Children  4 to 59 months,  N =127 | No follow-up. | [12] |
| Tanzania, 2009 | A positive *P. falciparum* microscopic blood smear with no fever or history of fever within the last two weeks, a normal white blood cell count, and no acute illness (bone fracture > 1 week old permitted) | Children  6 months to 9 years,  N=45 | No follow-up. | [13] |
| Western Uganda, 2009 | No malaria symptoms 2 weeks before and 1 week after day 0; axillary temperature ≤37.3 ◦C and a positive *P. falciparum* microscopic blood smear. | Children  3 months to 15 years, N=291 | Approximately 1 month. | [14] |
| Ghana, 2010 | Women with no clinical symptoms of malaria presenting at their third trimester for delivery but *P. falciparum positive* detected with Malaria Antigen ELISA kit. | Pregnant women  15 to 48 years, N=40 | No follow-up. | [15] |
| **South America** | | | | |
| Brazilian Amazon, 2002 | Individuals positive by microscopy, and/or positive by PCR; and individuals negative by microscopy who subsequently became positive by PCR. | All age groups,  N=172 | Follow-up to day 10 and 60. | [16] |
| Western Amazon, 2007 | Individuals who (a) did not report any febrile episodes two weeks before blood retrieval (b) did not present any symptoms on the day of blood retrieval and throughout the following 60 days; and sub-microscopical *P. falciparum* parasitemias (approximately 1-100 infected RBC per μl of erythrocytes). | Individuals between  3 to 49 years,  N=43 | Sixty days. | [17] |
| Brazilian Amazon, 2008 | *P. falciparum* infected persons without symptoms for 60 consecutive days confirmed as positive by polymerase chain reaction (PCR) analysis or a thick blood smear film. | Individuals with median age of 35.5 years,  N=304 | Sixty consecutive days. | [18] |
| Colombia, 2008 | Presence of microscopic asexual parasite stages of *P. falciparum, P. vivax* or *P. malarie* or of mixed infections in blood, which persisted for at least two weeks without causing any symptoms, or as the detection of parasite DNA by PCR on day 0 in people who remained asymptomatic during the follow-up period. | Individuals  2 to 78 years,  N=21 | Follow-up on days 14 and 28. | [19] |
| **Asia** | | | | |
| Papua, 2003 | No fever history or treatment for malaria within the past week, no clinical evidence of malaria or other infection, no diarrhoea, and no current pregnancy but both *P. falciparum* and *P. vivax* positive individuals | Adults  16 years and older,  N=105 | Supervised overnight at local health center, where clinical details were rechecked at interview in the evening and measurement of axillary temperature was repeated. A third axillary temperature was recorded the following morning. | [20] |
| Vietnam, 2008 | Body temperature *<*37.5 ◦C and presence of *P. falciparum* parasites detected by blood smear and/or PCR. | Individuals between  10 months to 72 years,  N=131 | No follow-up. | [21] |
| Indonesia, 2010 | Presence of asexual *P. falciparum* or *P. vivax* parasitemia in the absence of fever (temperature ≤37.9ºC) and of clinical signs or symptoms suggestive for malaria or another infectious disease. | Children  5 to 15 years,  N=381 | No follow-up. | [22] |

1. Amodu OK, Adeyemo AA, Olumese PE, Gbadegesin RA: **Intraleucocytic malaria pigment and clinical severity of malaria in children**. *Trans R Soc Trop Med Hyg* 1998, **92**:54-56.
2. Abdel-Latif MS, Dietz K, Issifou S, Kremsner PG, Klinkert MQ: **Antibodies to *Plasmodium falciparum* rifin proteins are associated with rapid parasite clearance and asymptomatic infections**. *Infect Immun* 2003, **71**:6229-6233.
3. Mombo LE, Ntoumi F, Bisseye C, Ossari S, Lu CY, Nagel RL, Krishnamoorthy R: **Human genetic polymorphisms and asymptomatic *Plasmodium falciparum* malaria in Gabonese schoolchildren**. *Am J Trop Med Hyg* 2003, **68**:186-190.
4. Matondo Maya DW, Mavoungou E, Deloron P, Theisen M, Ntoumi F: **Distribution of IgG subclass antibodies specific for *Plasmodium falciparum* glutamate-rich-protein molecule in sickle cell trait children with asymptomatic infections**. *Exp Parasitol* 2006, **112**:92-98.
5. Toure FS, Bisseye C, Mavoungou E: **Imbalanced distribution of *Plasmodium falciparum* EBA-175 genotypes related to clinical status in children from Bakoumba, Gabon**. *Clin Med Res* 2006, **4**:7-11.
6. Duarte J, Deshpande P, Guiyedi V, Mecheri S, Fesel C, Cazenave PA, Mishra GC, Kombila M, Pied S: **Total and functional parasite specific IgE responses in *Plasmodium falciparum*-infected patients exhibiting different clinical status**. *Malar J* 2007, **6**:1.
7. Mibei EK, Orago AS, Stoute JA: **Immune complex levels in children with severe *Plasmodium falciparum* malaria**. *Am J Trop Med Hyg* 2005, **72**:593-599.
8. Bejon P, Warimwe G, Mackintosh CL, Mackinnon MJ, Kinyanjui SM, Musyoki JN, Bull PC, Marsh K: **Analysis of immunity to febrile malaria in children that distinguishes immunity from lack of exposure**. *Infect Immun* 2009, **77**:1917-1923.
9. Farnert A, Williams TN, Mwangi TW, Ehlin A, Fegan G, Macharia A, Lowe BS, Montgomery SM, Marsh K: **Transmission-dependent tolerance to multiclonal *Plasmodium falciparum* infection**. *J Infect Dis* 2009, **200**:1166-1175.
10. Males S, Gaye O, Garcia A: **Long-term asymptomatic carriage of *Plasmodium falciparum* protects from malaria attacks: a prospective study among Senegalese children**. *Clin Infect Dis* 2008, **46**:516-522.
11. Bereczky S, Montgomery SM, Troye-Blomberg M, Rooth I, Shaw MA, Farnert A: **Elevated anti-malarial IgE in asymptomatic individuals is associated with reduced risk for subsequent clinical malaria**. *Int J Parasitol* 2004, **34**:935-942.
12. Rottmann M, Lavstsen T, Mugasa JP, Kaestli M, Jensen AT, Muller D, Theander T, Beck HP: **Differential expression of *var* gene groups is associated with morbidity caused by *Plasmodium falciparum* infection in Tanzanian children**. *Infect Immun* 2006, **74**:3904-3911.
13. Anstey NM, Weinberg JB, Wang Z, Mwaikambo ED, Duffy PE, Granger DL: **Effects of age and parasitemia on nitric oxide production/leukocyte nitric oxide synthase type 2 expression in asymptomatic, malaria-exposed children**. *Am J Trop Med Hyg* 1999, **61**:253-258.
14. Kiwanuka GN, Joshi H, Isharaza WK, Eschrich K: **Dynamics of *Plasmodium falciparum* alleles in children with normal haemoglobin and with sickle cell trait in western Uganda**. *Trans R Soc Trop Med Hyg* 2009, **103**:87-94.
15. Wilson NO, Bythwood T, Solomon W, Jolly P, Yatich N, Jiang Y, Shuaib F, Adjei AA, Anderson W, Stiles JK: **Elevated levels of IL-10 and G-CSF associated with asymptomatic malaria in pregnant women**. *Infect Dis Obstet Gynecol* 2010, **2010**.
16. Alves FP, Durlacher RR, Menezes MJ, Krieger H, Silva LH, Camargo EP: **High prevalence of asymptomatic *Plasmodium vivax* and *Plasmodium falciparum* infections in native Amazonian populations**. *Am J Trop Med Hyg* 2002, **66**:641-648.
17. dalla Martha RC, Tada MS, Ferreira RG, da Silva LH, Wunderlich G: **Microsatellite characterization of *Plasmodium falciparum* from symptomatic and non-symptomatic infections from the Western Amazon reveals the existence of non-symptomatic infection-associated genotypes**. *Mem Inst Oswaldo Cruz* 2007, **102**:293-298.
18. Leoratti FM, Farias L, Alves FP, Suarez-Mutis MC, Coura JR, Kalil J, Camargo EP, Moraes SL, Ramasawmy R: **Variants in the toll-like receptor signaling pathway and clinical outcomes of malaria**. *J Infect Dis* 2008, **198**:772-780.
19. Cucunuba ZM, Guerra AP, Rahirant SJ, Rivera JA, Cortes LJ, Nicholls RS: **Asymptomatic *Plasmodium spp*. infection in Tierralta, Colombia**. *Mem Inst Oswaldo Cruz* 2008, **103**:668-673.
20. Boutlis CS, Tjitra T, Maniboey H, Misukonis MA, Saunders JR, Suprianto S, Weinberg JB, Anstey NM: **Nitric Oxide Production and Mononuclear Cell Nitric Oxide Synthase activity in Malaria-Tolerant Papuan Adults**. *Infection and Immunity* 2003, **71**:3682–3689.
21. Maeno Y, Nakazawa S, Dao le D, Yamamoto N, Giang ND, Van Hanh T, Thuan le K, Taniguchi K: **A dried blood sample on filter paper is suitable for detecting *Plasmodium falciparum* gametocytes by reverse transcription polymerase chain reaction**. *Acta Trop* 2008, **107**:121-127.
22. de Mast Q, Syafruddin D, Keijmel S, Olde Riekerink T, Deky O, Asih PB, Swinkels DW, van der Ven AJ: **Increased serum hepcidin and alterations in blood iron parameters associated with asymptomatic *P. falciparum* and *P. vivax* malaria**. *Haematologica* 2010.
