# Supplementary material for: The complexities of malaria disease manifestations with a focus on asymptomatic malaria
Source: Malar J. 2012 Jan 31;11:29. doi: 10.1186/1475-2875-11-29 (PMC3342920; doi:10.1186/1475-2875-11-29)
Supplement: Additional file 2 — List of P. falciparum genes reported to be associated with different clinical outcomes of malaria. (DOC 78 kb). [file 1475-2875-11-29-S2.DOC]

**Additional file 2. List of *P. falciparum* genes reported to be associated with different clinical outcomes of malaria.**

| ***P. falciparum* gene name** | **Function** | **Implication in clinical outcome** | **Polymorphic allele /genotype/change in gene** | **Place of study conducted** | **References** |
| --- | --- | --- | --- | --- | --- |
| Merozoite Surface Protein 1 (MSP1) | Immune evasion | Associated with asymptomatic malaria  Associated with symptomatic malaria  Associated with severe malaria | K1 and MAD20  MAD 20  B-K1 | Nigeria  Ghana  French Guiana | [1]  [2]  [3] |
| Merozoite Surface Protein 2 (MSP2) | Immune evasion | More likely to be found in symptomatic and severe malaria | FC27 | Nigeria, Papua New Guinea, Ghana | [2,4-6] |
| Apical membrane Antigen 1 (AMA1) | Not well understood but it is believed to be involved in the process of invasion of host red blood cells | Associated with morbidity | Nucleotide sequence differences between symptomatic and asymptomatic malaria | Papua New Guinea | [7] |
| Erythrocyte Binding Antigen-175 (EBA-175) | Cell invasion | C-segment of the *eba-175* dimorphism on *P. falciparum merozoites* is associated with fatality in severe malaria rather than promoting disease progression from asymptomatic parasitaemia towards severe disease | CAMP(C-) and FCR-3 genotype | Tamale, Ghana | [8] |
| Knob Associated Histidine Rich Protein (KAHRP) | Required for knob formation | Associated with mild and severe malaria | Several different genotypes | India | [9] |
| Variant surface antigen (*var* gene family) | Virulence | High proportion of *var* group C transcripts was found in asymptomatic children and high proportion of *var* group B transcripts in children with clinical malaria (mild and severe);  the transcript abundances of var group A and B genes were higher for children with clinical malaria | Differential gene expression | Papua New Guinea,    Tanzania | [10]  [11] |
| *Plasmodium falciparum* Erythrocyte Membrane protein 1 (PfEMP1) encoded by *var* group A | Mediates cytoadherence to host cell endothelial receptors and antigenic variation | Associated with Severe malaria | Preferential expression of PfEMP1 encoded by group A *var* genes, up-regulation of group A  *var* genes;  PfEMP1-DBL1α | Ghana, Tanzania  Uganda | [12]  [13] |
| Microsatellite markers | Neutral | Some microsatellite loci are associated with symptomatic and certain others are associated with asymptomatic malaria | - | Brazil | [14] |
| *rif* (repetitive interspersed family) | RIFINs function as knob-accessory proteins | High levels of antirifin antibodies to rifin  proteins in asymptomatic parasitemic children | Differences in levels of antirifin antibodies between different clinical outcomes | Gabon | [15] |

1. Amodu OK, Adeyemo AA, Ayoola OO, Gbadegesin RA, Orimadegun AE, Akinsola AK, Olumese PE, Omotade OO: **Genetic diversity of the msp-1 locus and symptomatic malaria in south-west Nigeria**. *Acta Trop* 2005, **95**:226-232.
2. Ofosu-Okyere A, Mackinnon MJ, Sowa MP, Koram KA, Nkrumah F, Osei YD, Hill WG, Wilson MD, Arnot DE: **Novel *Plasmodium falciparum* clones and rising clone multiplicities are associated with the increase in malaria morbidity in Ghanaian children during the transition into the high transmission season**. *Parasitology* 2001, **123**:113-123.
3. Ariey F, Hommel D, Le Scanf C, Duchemin JB, Peneau C, Hulin A, Sarthou JL, Reynes JM, Fandeur T, Mercereau-Puijalon O: **Association of severe malaria with a specific *Plasmodium falciparum* genotype in French Guiana**. *J Infect Dis* 2001, **184**:237-241.
4. Amodu OK, Oyedeji SI, Ntoumi F, Orimadegun AE, Gbadegesin RA, Olumese PE, Omotade OO: **Complexity of the msp2 locus and the severity of childhood malaria, in south-western Nigeria**. *Ann Trop Med Parasitol* 2008, **102**:95-102.
5. Engelbrecht F, Felger I, Genton B, Alpers M, Beck HP: ***Plasmodium falciparum*: malaria morbidity is associated with specific merozoite surface antigen 2 genotypes**. *Exp Parasitol* 1995, **81**:90-96.
6. al-Yaman F, Genton B, Taraika J, Anders R, Alpers MP: **Cellular immunity to merozoite surface protein 2 (FC27 and 3D7) in Papua New Guinean children. Temporal variation and relation to clinical and parasitological status**. *Parasite Immunol* 1997, **19**:207-214.
7. Cortes A, Mellombo M, Mueller I, Benet A, Reeder JC, Anders RF: **Geographical structure of diversity and differences between symptomatic and asymptomatic infections for *Plasmodium falciparum* vaccine candidate AMA1**. *Infect Immun* 2003, **71**:1416-1426.
8. Cramer JP, Mockenhaupt FP, Mohl I, Dittrich S, Dietz E, Otchwemah RN, Ehrhardt S, Bienzle U, Jelinek T: **Allelic dimorphism of the erythrocyte binding antigen-175 (eba-175) gene of *Plasmodium falciparum* and severe malaria: Significant association of the C-segment with fatal outcome in Ghanaian children**. *Malar J* 2004, **3**:11.
9. Ranjit MR, Das A, Das BP, Das BN, Dash BP, Chhotray GP: **Distribution of *Plasmodium falciparum* genotypes in clinically mild and severe malaria cases in Orissa, India**. *Trans R Soc Trop Med Hyg* 2005, **99**:389-395.
10. Kaestli M, Cockburn IA, Cortes A, Baea K, Rowe JA, Beck HP: **Virulence of malaria is associated with differential expression of *Plasmodium falciparum* *var* gene subgroups in a case-control study**. *J Infect Dis* 2006, **193**:1567-1574.
11. Rottmann M, Lavstsen T, Mugasa JP, Kaestli M, Jensen AT, Muller D, Theander T, Beck HP: **Differential expression of *var* gene groups is associated with morbidity caused by *Plasmodium falciparum* infection in Tanzanian children**. *Infect Immun* 2006, **74**:3904-3911.
12. Jensen AT, Magistrado P, Sharp S, Joergensen L, Lavstsen T, Chiucchiuini A, Salanti A, Vestergaard LS, Lusingu JP, Hermsen R, Sauerwein R, Christensen J, Nielsen MA, Hviid L, Sutherland C, Staalsoe T, Theander TG: ***Plasmodium falciparum* associated with severe childhood malaria preferentially expresses PfEMP1 encoded by group A var genes**. *J Exp Med* 2004, **199**:1179-1190.
13. Normark J, Nilsson D, Ribacke U, Winter G, Moll K, Wheelock CE, Bayarugaba J, Kironde F, Egwang TG, Chen Q, Andersson B, Wahlgren M: **PfEMP1-DBL1alpha amino acid motifs in severe disease states of *Plasmodium falciparum* malaria**. *Proc Natl Acad Sci U S A* 2007, **104**:15835-15840.
14. dalla Martha RC, Tada MS, Ferreira RG, da Silva LH, Wunderlich G: **Microsatellite characterization of *Plasmodium falciparum* from symptomatic and non-symptomatic infections from the Western Amazon reveals the existence of non-symptomatic infection-associated genotypes**. *Mem Inst Oswaldo Cruz* 2007, **102**:293-298.
15. Abdel-Latif MS, Dietz K, Issifou S, Kremsner PG, Klinkert MQ: **Antibodies to *Plasmodium falciparum* rifin proteins are associated with rapid parasite clearance and asymptomatic infections**. *Infect Immun* 2003, **71**:6229-6233.
