# Supplementary material for: The complexities of malaria disease manifestations with a focus on asymptomatic malaria
Source: Malar J. 2012 Jan 31;11:29. doi: 10.1186/1475-2875-11-29 (PMC3342920; doi:10.1186/1475-2875-11-29)
Supplement: Additional file 3 — List of human genes reported to be associated with different clinical outcomes of malaria. A. Human gene polymorphisms B. Human blood disorders. [file 1475-2875-11-29-S3.DOC]

**Additional file 3.** List of human genes reported to be associated with different clinical outcomes of malaria.

A. Human gene polymorphisms B. Human blood disorders.

A.

| **Human gene name** | **Function** | **Implication in clinical outcome/ Association study** | **Polymorphic allele /genotype/ change in gene** | **Place of study conducted** | **References** |
| --- | --- | --- | --- | --- | --- |
| Fcγ Receptor II A (FcγRIIA) | Regulation of IgG isotype level | ↑IgG2 levels in asymptomatic  subjects;  ↓parasitaemia | H131  R131 | Amazon Basin of Brazil  Western Kenya | [1]  [2] |
| Fcγ Receptor II B (FcγRIIB) | Regulation of IgG isotype level | Susceptibility to severe malaria | Deficiency of FcγRIIB | Studied in mice model | [3] |
| Tumor Necrosis Factor-α (TNF-α) | Involved in iinflammatory and immune responses and plays an important role in the pathogenesis of many infectious diseases | Protection against clinical malaria  Increased susceptibility to severe malaria | TNF-238A  TNF-308A  TNF -1031C and -863A alleles | Gambia, Gabon  India | [4-5]  [6]  [7] |
| Human Leucocyte Antigen (HLA) | Antigen presentation and recognition | Protection from severe malaria | HLA-B53 | Africa | [8-10] |
| Interferon Gamma Receptor (IFNGR) | Key mediator of anti-parasitic immune effector mechanisms | Protection against cerebral malaria, severe malaria and fatal outcome | IFNGR1-56T/C, IFNGR1-470 in/del | Gambia | [11-12] |
| Interferon Regulatory Factor (IRF1) | Transcription factor which is a mediator of INFγ activity | Associated with severe malaria | IRF1rs10065633 C | West Africa | [13] |
| Cluster for Differentiation 40 Ligand (CD40L) | Involved in B cell proliferation, antigen presenting cell activation, and Ig isotype switching | Significant reduction in risk for severe malaria was associated with males hemizygous for CD40L-726C | CD40L-726C | Gambia | [14] |
| Mannose Binding Lectin (MBL2) | activation of the complement system and opsonization of pathogenic microorganisms | Asymptomatic : susceptibility to infection and parasitaemia control | *LYPA/LYPA* genotype | Gabon | [15] |
| Interleukin 1 (IL1) | Pro-inflamatory cytokine which is a potent endogenous pyrogen and inducer of the acute phase response | Involved in malaria susceptibility | Interleukin-1 alpha +4845 G→ T; interleukin-1 beta +3953 C → T | Gambia | [16] |
| Interleukin 4 (IL4) | Modulation of antibodies production | Significant inter-ethnic difference in allele and genotype frequency in asymptomatic subjects;  Increase total IgE in children with severe malaria | IL-4 -590 C/T  IL-4-589C/T | Mali  Burkina Faso, West Africa | [17]  [18] |
| Variants in Toll Like Receptor (TLR)-signaling pathway | Recognition of invading pathogens through distinct  pathogen- associated molecular patterns; innate immunity of the host. | High parasitaemia;  Mild malaria | *TLR-9 -1486T/C*  *TLR-1* and *TLR-6* variants | Amazonian region of Brazil | [19] |
| Intercellular Cell Adhesion Molecule (ICAM1) | Receptor for infected erythrocytes on endothelium | Predispose to severe malaria | ICAM-1^kilifi^ | Africa | [20] |
| Cluster for Differentiation 36 (CD36) | Receptor for adhesion of infected erythrocytes | Protection against severe malaria by reducing parasite sequestration | Cd36-T188G | Kenya | [21] |
| Platelet Endothelial Cell Adhesion Molecule 1 (PECAM1/CD31) | Removal of aged neutrophils, endothelial cell receptor of *P. falciparum*–infected erythrocytes | Associated with cerebral malaria | 125 V/V 563 N/N genotype | Papua New Guinea, Kenya | [22-23] |
| Complement Receptor 1 (CR1) | Complement activation and clearance of immune complexes | Protect against severe malaria | CR1 deficiency | Africa, Papua New Guinea, Mali, western Kenya | [24-27] |
| Glucose 6 Phosphate Dehydrogenase (G6PD) | Catalyzes the initial step of the pentose phosphate pathway and produces reduced glutathione and control oxidative stress | Protection against uncomplicated and severe malaria | Several SNPs | Africa, Mali | [28-33] |
| Nitric Oxide Synthase 2 (NOS2) | Mediator of immunity and modulate production of NO | Associated with severe malaria;  Protects heterozygous carriers against severe malaria;  Associated with protection from symptomatic malaria and protection from severe malarial anaemia | CCTTT microsatellite repeat;  NOS2-G954C  -1173 C-->T in the NOS2 promoter | Thailand  Africa  Tanzania and Kenya | [34]  [35]  [36] |

B.

| **Human Blood Polymorphism/ Disorder** | **Implication in clinical outcome/ Association study** | **Polymorphic allele /genotype/ change in gene** | **Place of study conducted** | **References** |
| --- | --- | --- | --- | --- |
| ABO blood group | Protects against severe malaria, Asymptomatic | Blood group O | Gabon, Mali, Zimbabwe | [37-42] |
| Haptoglobin (HP) | Associated with susceptibility to falciparum malaria and the development of severe complications | Hp1-1, A-61C SNP | Sudan, Ghana, Gambia | [43-45] |
| Hemoglobin (HB) variants | HBS associated with protection against mild malaria attacks  HBC protects against severe malaria  HBE protects against severe malaria | β6Glu→Val  β6Glu→Lys  β26Glu→Lys | Senegal  Mali, Burkina Faso  Thailand | [46]  [47-48]  [49] |
| Thalassaemia | Protects against severe malaria | α- and β- thalassaemia | Papua New Guinea; Ghana | [50-52] |
| Ovalocytosis | Protection against severe malaria, specifically against the cerebral form | Deletion of the erythrocyte membrane band 3 gene | South-East Asia and Papua New Guinea | [53-54] |

1. Leoratti FM, Durlacher RR, Lacerda MV, Alecrim MG, Ferreira AW, Sanchez MC, Moraes SL: **Pattern of humoral immune response to *Plasmodium falciparum* blood stages in individuals presenting different clinical expressions of malaria**. *Malar J* 2008, **7**:186.
2. Shi YP, Nahlen BL, Kariuki S, Urdahl KB, McElroy PD, Roberts JM, Lal AA: **Fcgamma receptor IIa (CD32) polymorphism is associated with protection of infants against high-density *Plasmodium falciparum* infection. VII. Asembo Bay Cohort Project**. *J Infect Dis* 2001, **184**:107-111.
3. Clatworthy MR, Willcocks L, Urban B, Langhorne J, Williams TN, Peshu N, Watkins NA, Floto RA, Smith KG: **Systemic lupus erythematosus-associated defects in the inhibitory receptor FcgammaRIIb reduce susceptibility to malaria**. *Proc Natl Acad Sci U S A* 2007, **104**:7169-7174.
4. McGuire W, Hill AV, Allsopp CE, Greenwood BM, Kwiatkowski D: **Variation in the TNF-alpha promoter region associated with susceptibility to cerebral malaria**. *Nature* 1994, **371**:508-510.
5. Mombo LE, Ntoumi F, Bisseye C, Ossari S, Lu CY, Nagel RL, Krishnamoorthy R: **Human genetic polymorphisms and asymptomatic *Plasmodium falciparum* malaria in Gabonese schoolchildren**. *Am J Trop Med Hyg* 2003, **68**:186-190.
6. McGuire W, Knight JC, Hill AV, Allsopp CE, Greenwood BM, Kwiatkowski D: **Severe malarial anemia and cerebral malaria are associated with different tumor necrosis factor promoter alleles**. *J Infect Dis* 1999, **179**:287-290.
7. Sinha S, Mishra SK, Sharma S, Patibandla PK, Mallick PK, Sharma SK, Mohanty S, Pati SS, Mishra SK, Ramteke BK, Bhatt R, Joshi H, Dash AP, Ahuja RC, Awasthi S, Venkatesh V, Habib S: **Polymorphisms of TNF-enhancer and gene for FcgammaRIIa correlate with the severity of falciparum malaria in the ethnically diverse Indian population**. *Malar J* 2008, **7**:13.
8. Hill AV, Elvin J, Willis AC, Aidoo M, Allsopp CE, Gotch FM, Gao XM, Takiguchi M, Greenwood BM, Townsend AR, McMichael AJ, Whittle HC: **Molecular analysis of the association of HLA-B53 and resistance to severe malaria**. *Nature* 1992, **360**:434-439.
9. Bennett S, Allen SJ, Olerup O, Jackson DJ, Wheeler JG, Rowe PA, Riley EM, Greenwood BM: **Human leucocyte antigen (HLA) and malaria morbidity in a Gambian community**. *Trans R Soc Trop Med Hyg* 1993, **87**:286-287.
10. Gilbert SC, Plebanski M, Gupta S, Morris J, Cox M, Aidoo M, Kwiatkowski D, Greenwood BM, Whittle HC, Hill AV: **Association of malaria parasite population structure, HLA, and immunological antagonism**. *Science* 1998, **279**:1173-1177.
11. Koch O, Kwiatkowski DP, Udalova IA: **Context-specific functional effects of IFNGR1 promoter polymorphism**. *Hum Mol Genet* 2006, **15**:1475-1481.
12. Koch O, Awomoyi A, Usen S, Jallow M, Richardson A, Hull J, Pinder M, Newport M, Kwiatkowski D: **IFNGR1 gene promoter polymorphisms and susceptibility to cerebral malaria**. *J Infect Dis* 2002, **185**:1684-1687.
13. Mangano VD, Luoni G, Rockett KA, Sirima BS, Konate A, Forton J, Clark TG, Bancone G, Sadighi Akha E, Kwiatkowski DP, Modiano D: **Interferon regulatory factor-1 polymorphisms are associated with the control of *Plasmodium falciparum* infection**. *Genes Immun* 2008, **9**:122-129.
14. Sabeti P, Usen S, Farhadian S, Jallow M, Doherty T, Newport M, Pinder M, Ward R, Kwiatkowski D: **CD40L association with protection from severe malaria**. *Genes Immun* 2002, **3**:286-291.
15. Boldt AB, Messias-Reason IJ, Lell B, Issifou S, Pedroso ML, Kremsner PG, Kun JF: **Haplotype specific-sequencing reveals MBL2 association with asymptomatic *Plasmodium falciparum* infection**. *Malar J* 2009, **8**:97.
16. Walley AJ, Aucan C, Kwiatkowski D, Hill AV: **Interleukin-1 gene cluster polymorphisms and susceptibility to clinical malaria in a Gambian case-control study**. *Eur J Hum Genet* 2004, **12**:132-138.
17. Vafa M, Maiga B, Berzins K, Hayano M, Bereczky S, Dolo A, Daou M, Arama C, Kouriba B, Farnert A, Doumbo OK, Troye-Blomberg M: **Associations between the IL-4 -590 T allele and *Plasmodium falciparum* infection prevalence in asymptomatic Fulani of Mali**. *Microbes Infect* 2007, **9**:1043-1048.
18. Verra F, Luoni G, Calissano C, Troye-Blomberg M, Perlmann P, Perlmann H, Arca B, Sirima BS, Konate A, Coluzzi M, Kwiatkowski D, Modiano D: **IL4-589C/T polymorphism and IgE levels in severe malaria**. *Acta Trop* 2004, **90**:205-209.
19. Leoratti FM, Farias L, Alves FP, Suarez-Mutis MC, Coura JR, Kalil J, Camargo EP, Moraes SL, Ramasawmy R: **Variants in the toll-like receptor signaling pathway and clinical outcomes of malaria**. *J Infect Dis* 2008, **198**:772-780.
20. Fernandez-Reyes D, Craig AG, Kyes SA, Peshu N, Snow RW, Berendt AR, Marsh K, Newbold CI: **A high frequency African coding polymorphism in the N-terminal domain of ICAM-1 predisposing to cerebral malaria in Kenya**. *Hum Mol Genet* 1997, **6**:1357-1360.
21. Pain A, Urban BC, Kai O, Casals-Pascual C, Shafi J, Marsh K, Roberts DJ: **A non-sense mutation in Cd36 gene is associated with protection from severe malaria**. *Lancet* 2001, **357**:1502-1503.
22. Kikuchi M, Looareesuwan S, Ubalee R, Tasanor O, Suzuki F, Wattanagoon Y, Na-Bangchang K, Kimura A, Aikawa M, Hirayama K: **Association of adhesion molecule PECAM-1/CD31 polymorphism with susceptibility to cerebral malaria in Thais**. *Parasitol Int* 2001, **50**:235-239.
23. Casals-Pascual C, Allen S, Allen A, Kai O, Lowe B, Pain A, Roberts DJ: **Short report: codon 125 polymorphism of CD31 and susceptibility to malaria**. *Am J Trop Med Hyg* 2001, **65**:736-737.
24. Cockburn IA, Mackinnon MJ, O'Donnell A, Allen SJ, Moulds JM, Baisor M, Bockarie M, Reeder JC, Rowe JA: **A human complement receptor 1 polymorphism that reduces *Plasmodium falciparum* rosetting confers protection against severe malaria**. *Proc Natl Acad Sci U S A* 2004, **101**:272-277.
25. Rowe JA, Moulds JM, Newbold CI, Miller LH: ***P. falciparum* rosetting mediated by a parasite-variant erythrocyte membrane protein and complement-receptor 1**. *Nature* 1997, **388**:292-295.
26. Moulds JM, Kassambara L, Middleton JJ, Baby M, Sagara I, Guindo A, Coulibaly S, Yalcouye D, Diallo DA, Miller L, Doumbo O: **Identification of complement receptor one (CR1) polymorphisms in west Africa**. *Genes Immun* 2000, **1**:325-329.
27. Waitumbi JN, Opollo MO, Muga RO, Misore AO, Stoute JA: **Red cell surface changes and erythrophagocytosis in children with severe *Plasmodium falciparum* anemia**. *Blood* 2000, **95**:1481-1486.
28. Ruwende C, Khoo SC, Snow RW, Yates SN, Kwiatkowski D, Gupta S, Warn P, Allsopp CE, Gilbert SC, Peschu N, Newbold CI, Greenwood BM, Marsh K, Hill AVS: **Natural selection of hemi- and heterozygotes for G6PD deficiency in Africa by resistance to severe malaria**. *Nature* 1995, **376**:246-249.
29. Usanga EA, Luzzatto L: **Adaptation of *Plasmodium falciparum* to glucose 6-phosphate dehydrogenase-deficient host red cells by production of parasite-encoded enzyme**. *Nature* 1985, **313**:793-795.
30. Friedman MJ: **Oxidant damage mediates variant red cell resistance to malaria**. *Nature* 1979, **280**:245-247.
31. Roth EF, Jr., Raventos-Suarez C, Rinaldi A, Nagel RL: **Glucose-6-phosphate dehydrogenase deficiency inhibits in vitro growth of *Plasmodium falciparum***. *Proc Natl Acad Sci U S A* 1983, **80**:298-299.
32. Cappadoro M, Giribaldi G, O'Brien E, Turrini F, Mannu F, Ulliers D, Simula G, Luzzatto L, Arese P: **Early phagocytosis of glucose-6-phosphate dehydrogenase (G6PD)-deficient erythrocytes parasitized by *Plasmodium falciparum* may explain malaria protection in G6PD deficiency**. *Blood* 1998, **92**:2527-2534.
33. Guindo A, Fairhurst RM, Doumbo OK, Wellems TE, Diallo DA: **X-linked G6PD deficiency protects hemizygous males but not heterozygous females against severe malaria**. *PLoS Med* 2007, **4**:e66.
34. Ohashi J, Naka I, Patarapotikul J, Hananantachai H, Looareesuwan S, Tokunaga K: **Significant association of longer forms of CCTTT microsatellite repeat in the inducible nitric oxide synthase promoter with severe malaria in Thailand**. *J Infect Dis* 2002, **186**:578-581.
35. Kun JF, Mordmuller B, Perkins DJ, May J, Mercereau-Puijalon O, Alpers M, Weinberg JB, Kremsner PG: **Nitric oxide synthase 2(Lambarene) (G-954C), increased nitric oxide production, and protection against malaria**. *J Infect Dis* 2001, **184**:330-336.
36. Hobbs MR, Udhayakumar V, Levesque MC, Booth J, Roberts JM, Tkachuk AN, Pole A, Coon H, Kariuki S, Nahlen BL, Mwaikambo ED, Lal AL, Granger DL, Anstey NM, Weinberg JB: **A new NOS2 promoter polymorphism associated with increased nitric oxide production and protection from severe malaria in Tanzanian and Kenyan children**. *Lancet* 2002, **360**:1468-1475.
37. Mombo LE, Ntoumi F, Bisseye C, Ossari S, Lu CY, Nagel RL, Krishnamoorthy R: **Human genetic polymorphisms and asymptomatic *Plasmodium falciparum* malaria in Gabonese schoolchildren**. *Am J Trop Med Hyg* 2003, **68**:186-190.
38. Rowe JA, Handel IG, Thera MA, Deans AM, Lyke KE, Kone A, Diallo DA, Raza A, Kai O, Marsh K, Plowe CV, Doumbo OK, Moulds JM: **Blood group O protects against severe *Plasmodium falciparum* malaria through the mechanism of reduced rosetting**. *Proc Natl Acad Sci U S A* 2007, **104**:17471-17476.
39. Fischer PR, Boone P: **Short report: severe malaria associated with blood group**. *Am J Trop Med Hyg* 1998, **58**:122-123.
40. Lell B, May J, Schmidt-Ott RJ, Lehman LG, Luckner D, Greve B, Matousek P, Schmid D, Herbich K, Mockenhaupt FP, Meyer CG, Bienzle U, Kremsner PG: **The role of red blood cell polymorphisms in resistance and susceptibility to malaria**. *Clin Infect Dis* 1999, **28**:794-799.
41. Barragan A, Kremsner PG, Wahlgren M, Carlson J: **Blood group A antigen is a coreceptor in *Plasmodium falciparum* rosetting**. *Infect Immun* 2000, **68**:2971-2975.
42. Pare G, Chasman DI, Kellogg M, Zee RY, Rifai N, Badola S, Miletich JP, Ridker PM: **Novel association of ABO histo-blood group antigen with soluble ICAM-1: results of a genome-wide association study of 6,578 women**. *PLoS Genet* 2008, **4**:e1000118.
43. Elagib AA, Kider AO, Akerstrom B, Elbashir MI: **Association of the haptoglobin phenotype (1-1) with falciparum malaria in Sudan**. *Trans R Soc Trop Med Hyg* 1998, **92**:309-311.
44. Quaye IK, Ekuban FA, Goka BQ, Adabayeri V, Kurtzhals JA, Gyan B, Ankrah NA, Hviid L, Akanmori BD: **Haptoglobin 1-1 is associated with susceptibility to severe *Plasmodium falciparum* malaria**. *Trans R Soc Trop Med Hyg* 2000, **94**:216-219.
45. Cox SE, Doherty C, Atkinson SH, Nweneka CV, Fulford AJ, Ghattas H, Rockett KA, Kwiatkowski DP, Prentice AM: **Haplotype association between haptoglobin (Hp2) and Hp promoter SNP (A-61C) may explain previous controversy of haptoglobin and malaria protection**. *PLoS One* 2007, **2**:e362.
46. Migot-Nabias F, Pelleau S, Watier L, Guitard J, Toly C, De Araujo C, Ngom MI, Chevillard C, Gaye O, Garcia A: **Red blood cell polymorphisms in relation to *Plasmodium falciparum* asymptomatic parasite densities and morbidity in Senegal**. *Microbes Infect* 2006, **8**:2352-2358.
47. Modiano D, Luoni G, Sirima BS, Simpore J, Verra F, Konate A, Rastrelli E, Olivieri A, Calissano C, Paganotti GM, D'Urbano L, Sanou I, Sawadogo A, Modiano G, Coluzzi M: **Haemoglobin C protects against clinical *Plasmodium falciparum* malaria**. *Nature* 2001, **414**:305-308.
48. Agarwal A, Guindo A, Cissoko Y, Taylor JG, Coulibaly D, Kone A, Kayentao K, Djimde A, Plowe CV, Doumbo O, Wellems TE, Diallo D: **Hemoglobin C associated with protection from severe malaria in the Dogon of Mali, a West African population with a low prevalence of hemoglobin S**. *Blood* 2000, **96**:2358-2363.
49. Hutagalung R, Wilairatana P, Looareesuwan S, Brittenham GM, Aikawa M, Gordeuk VR: **Influence of hemoglobin E trait on the severity of falciparum malaria**. *J Infect Dis* 1999, **179**:283-286.
50. Allen SJ, O'Donnell A, Alexander ND, Alpers MP, Peto TE, Clegg JB, Weatherall DJ: **alpha+-Thalassemia protects children against disease caused by other infections as well as malaria**. *Proc Natl Acad Sci U S A* 1997, **94**:14736-14741.
51. May J, Evans JA, Timmann C, Ehmen C, Busch W, Thye T, Agbenyega T, Horstmann RD: **Hemoglobin variants and disease manifestations in severe falciparum malaria**. *Jama* 2007, **297**:2220-2226.
52. Luzzi GA, Merry AH, Newbold CI, Marsh K, Pasvol G: **Protection by alpha-thalassaemia against *Plasmodium falciparum* malaria: modified surface antigen expression rather than impaired growth or cytoadherence**. *Immunol Lett* 1991, **30**:233-240.
53. Genton B, al-Yaman F, Mgone CS, Alexander N, Paniu MM, Alpers MP, Mokela D: **Ovalocytosis and cerebral malaria**. *Nature* 1995, **378**:564-565.
54. Allen SJ, O'Donnell A, Alexander ND, Mgone CS, Peto TE, Clegg JB, Alpers MP, Weatherall DJ: **Prevention of cerebral malaria in children in Papua New Guinea by southeast Asian ovalocytosis band 3**. *Am J Trop Med Hyg* 1999, **60**:1056-1060.
